# Analysis of Antibiotics in Bivalves by Ultra-High Performance Liquid Chromatography–Quadrupole Time-of-Flight Mass Spectrometry

**DOI:** 10.3390/antibiotics12050913

**Published:** 2023-05-15

**Authors:** André M. P. T. Pereira, Andreia Freitas, Angelina Pena, Liliana J. G. Silva

**Affiliations:** 1 LAQV, REQUIMTE, Laboratory of Bromatology and Pharmacognosy, Faculty of Pharmacy, University of Coimbra, Polo III, Azinhaga de Sta Comba, 3000-548 Coimbra, Portugal; andrepereira@ff.uc.pt (A.M.P.T.P.); apena@ci.uc.pt (A.P.); 2 National Institute for Agricultural and Veterinary Research (INIAV), I.P., Av. da República, Quinta do Marquês, 2780-157 Oeiras, Portugal; andreia.freitas@iniav.pt; 3 Associated Laboratory for Green Chemistry of the Network of Chemistry and Technology, LAQV/REQUIMTE, R. D. Manuel II, Apartado 55142, 4051-401 Porto, Portugal

**Keywords:** bivalves, antibiotics, food safety, environment contamination, biomonitoring, UHPLC-ToF-MS, validation

## Abstract

The presence of pharmaceuticals in aquatic ecosystems mostly originates from wastewater treatment plants (WWTPs) and such a situation can be responsible for significant negative impacts on natural ecosystems, such as estuarine and coastal areas. Bioaccumulation of pharmaceuticals, namely antibiotics, in exposed organisms is known to have remarkable effects on different trophic levels of non-target organisms such as algae, invertebrates and vertebrates, including the emergence of bacterial resistance. Bivalves are a highly appreciated seafood product, as they are fed by filtering water, and can bioconcentrate chemicals, being ideal for biomonitoring environmental health hazards in coastal and estuarine ecosystems. To use this sentinel species, an analytical strategy was developed to be used in accessing antibiotics, from human and veterinary medicine, and evaluate their occurrence as emerging pollutants in aquatic environments. The optimized analytical method was fully validated according to the European requirements defined by the Commission Implementing Regulation 2021/808. The validation comprised the following parameters: specificity, selectivity, precision, recovery, ruggedness, linearity, and the decision limit CCα, as well as the limit of detection (LoD) and limit of quantification (LoQ). The method was validated for 43 antibiotics to allow their quantification in both contexts, environmental biomonitoring and food safety.

## 1. Introduction

**Figure 1:**
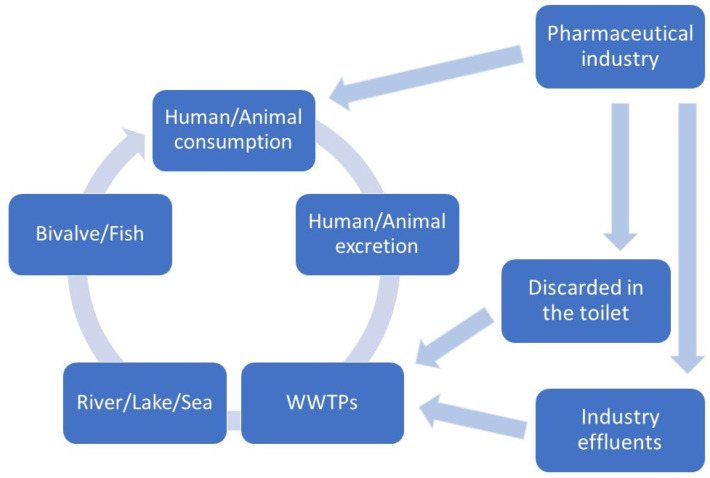
Schematic representation of pharmaceuticals, including antibiotics, path in the environment.

The presence of pharmaceuticals in aquatic ecosystems has been reported, originating both from diffuse and point sources (Figure 1). Wastewater treatment plants (WWTPs) are considered their main entry points, due to the inefficiency of the applied water treatments, with consequent impacts on natural ecosystems, such as estuarine and coastal areas [1].

Pharmaceuticals have a clear mode of action in target organisms and, given that fish and invertebrates share drug targets with humans, it would be expected that they would respond similarly. However, when non-target species are exposed, unknown effects and potential risks arise, even at the ng L^−1^ level [2]. Moreover, pharmaceuticals bioaccumulate in exposed organisms’ tissues, where sources of human sewage pollution proliferate [3,4,5], having remarkable effects on different trophic levels of non-target organisms, such as algae, invertebrates and vertebrates [2,6].

Regarding antibiotics, besides their direct toxicological risks, concern has been raised because they promote the emergence of resistant bacteria and the subsequent development of more resistant and virulent pathogens. These bacterial resistances, through horizontal gene transfer, may end up in human pathogens, raising questions about human health and ecosystem stability. In addition, scientific evidence suggests that resistance might be acquired faster in the aquatic environment when compared to the terrestrial one [2]. Therefore, the major risk regarding the presence of pharmaceutical residues in the aquatic environment is the emergence of bacterial resistance [7].

In recent years, antibiotics usage has increased globally, in both human and veterinary medicine. This led to their accumulation in the environment to such an extent that they are included in the category of contaminants of emerging concern. For this reason, some of them have been included in monitoring lists of potential pollutants by competent authorities to limit their presence in surface waters and to determine the risk to the aquatic environments [8]. At the European level, a watch list, under the Water Framework Directive, states that certain pollutants, including antibiotics, must be regularly monitored in surface waters. This list includes the macrolide antibiotics erythromycin, clarithromycin and azithromycin, as well as amoxicillin and ciprofloxacin [9].

Bivalves are sessile filter feeders that bioconcentrate chemicals, being ideal organisms as indicators of environmental health hazards. Moreover, bivalves are key components of coastal and estuarine ecosystems. They are usually biomass dominant and highly productive, playing a central role in the food web-linking primary producers and epibenthic consumers and providing essential ecosystem services. They are also economically valuable as a food resource, being harvested for human consumption for centuries, and more recently, produced in aquaculture to supply the growing consumption demand [10,11].

Biological matrices, due to their complexity, generally require long sample preparation. There are several extraction techniques used, mostly based on solid phase extraction (SPE) and pressurized liquid extraction (PLE), followed by an instrumental analysis based on liquid chromatography (LC) coupled to tandem mass spectrometry (MS/MS), in order to attain selectivity, sensitivity and robustness—mandatory in the quantitative analysis of trace-level residues in such complex matrices. In 2015, an analytical method was developed and validated to simultaneously determine 23 pharmaceuticals and some of their main metabolites, including the antibiotics ronidazole, metronidazole, dimetridazole, sulfamethoxazole, N-acetylsulfamethoxazole, azithromycin and erythromycin, in *Crassostrea gigas*, *Mytilus galloprovincialis* and *Chamelea gallina*. The extraction with both pressurized liquid (PLE) and SPE followed by UHPLC-MS/MS allowed for limits of detection (LoDs) ranging from 0.01 ng g^−1^ for ronidazole and 0.80 ng g^−1^ for metronidazole and limits of detection (LoQs) varying between 0.02 ng g^−1^ for azithromycin in the Pacific oyster and 3.70 ng g^−1^ for erythromycin in the Mediterranean mussel [12]. In another study, also aiming to analyze pharmaceuticals in bivalves (*Mytilus* spp.), including an antibiotic, trimethoprim, the extraction was done by PLE and SPE following LC-MS/MS. The lower LoQ was obtained for the trimethoprim (4 ng g^−1^) [13].

This study aimed to validate an analytical methodology, specific for antibiotics; to assess their presence in bivalves, an excellent sentinel species for these emerging pollutants and a tool for environmental monitoring in aquatic environments; and to evaluate the risk for human health following consumption of these filter feeders.

## 2. Results and Discussion

Although considered to be an effective choice as a bioindicator for environmental contamination, limited previous studies reporting methods for assessment of pharmaceutical active compounds in bivalve matrices are available. The use of such organisms has been reported mainly for other groups of pollutants such as metals, persistent organic pollutants polychlorinated biphenyl (PCBs), organochlorine pesticides (OCPs) and polycyclic aromatic hydrocarbons (PAHs) or microplastics [14,15,16,17,18]. Methods presented in the literature are almost related to the use of low-resolution mass spectrometry (such as LC-MS/MS) [8], which can be limited in terms of sensitivity for a high number of compounds analyzed at once. The use of a time-of-flight mass spectrometer, as high-resolution detection equipment, allows the addition of unlimited compounds without compromising the sensitivity of the results, and the possibility of a retrospective analysis. This is an important feature when it comes to assessing contaminants in the environment. In the future, the results can be re-visited to analyze untargeted compounds at the time of the first analysis.

The developed method was based on previous methods optimized to detect and quantify antibiotics in muscle [19]. After testing the method for the 43 target antibiotics from different families (beta-lactams, cephalosporins, macrolides, sulphonamides, quinolones, tetracyclines and trimethoprim), the next step was the validation in accordance with the European regulation in place (CIR 2021/808) [20] and the MRLs established for muscle from any food-producing species (EC Reg. n°37/2010) [21]. Since no specific MRLs are set for bivalve matrices, the MRL used for validation purposes was defined for muscle of all producing animals or, when such a situation is not described, for the lower MRL set for muscle. In practice, and since the matrix analyzed is the whole homogenized bivalve, the muscle matrix is the most similar.

### 2.1. Method Validation

To assess the European requirements and to prove that the method is suitable for its intended purpose, the following parameters were evaluated during the validation process: specificity, selectivity, precision, recovery, ruggedness, linearity and the decision limit CCα defined as the decision limit for confirmation methods. Additionally, in order to access the method limits for bivalves, the limit of detection (LoD) and limit of quantification (LoQ) were also calculated, as described in the ICH guidelines [22].The uncertainty (U) of the method was also evaluated based on the inter-day precision. As stated in the CIR 2021/808 [20], the within-laboratory reproducibility can be used to assess uncertainty since it is determined with variation of relevant factors that can influence the analysis. All results obtained during the validation are summarized in Table 1.

All parameters were evaluated using the relative intensities obtained from the ratio of the area of each antibiotic and the IS. The purpose of using an IS is to correct for possible sample preparation and detection-related variations. Sulfameter showed to be a very efficient and versatile IS since it provided good linearity-matrix-match calibration curves with a coefficient of correlation higher than 0.99 for all antibiotics, except epi-tetracycline with R^2^ > 0.97. Considering that the calibration curves are obtained from spiked samples and the potential matrix effects that the bivalve can provide, a coefficient of correlation above 0.95 is acceptable.

Antibiotics, being allowed substances to be used in veterinary medicine practice, even in animal production, have MRLs defined for muscle and those values were used to calculate the decision limit CCα for each compound, according to Equation (1).

Equation (1):

(1) $$\mathrm{CC}\alpha \;=\;\mathrm{MRL}\;+1.64\times \sigma_{\mathrm{MRL}}$$

In the previous equation, $\sigma_{\mathrm{MRL}}$ defines the reproducibility obtained after analyzing 20 blank bivalve samples spiked at the MRL concentration. In terms of food safety, a result above CCα leads to the conclusion that the product is non-compliant, and the product is considered to be not safe for consumers.

Equations (2) and (3) give the formulas of LoD and LoQ calculation, respectively, where σ is the standard deviation obtained by the analysis of 20 blank bivalve samples and S is the calibration curve slope.

Equation (2):

(2) $$\mathrm{LoD}=\frac{3.3\times \sigma }{S}$$

Equation (3):

(3) $$\mathrm{LoQ}=\frac{10\times \sigma }{S}$$

In terms of limits, the lowest values were achieved for cefalonium, with LoD 3.05 μg kg^−1^ and LoQ 9.70 μg kg^−1^. On the other hand, the highest values were achieved for oxacillin, with LoD 57.9 μg kg^−1^ and LoQ 175.0 μg kg^−1^. In addition, these values are clearly below the established MRL of 300 μg kg^−1^ for muscle from any food-producing species.

**Figure 2:**
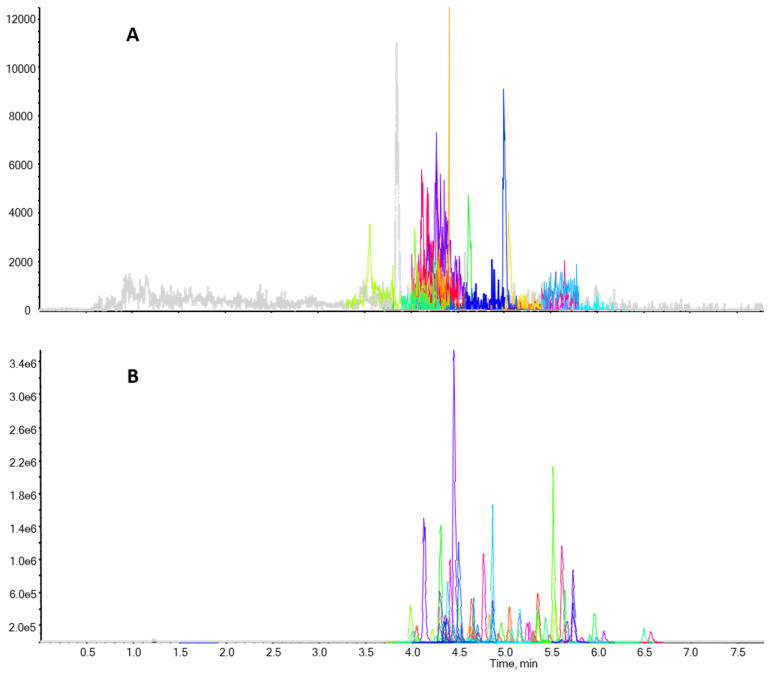
UHPLC-ToF-MS chromatograms: (**A**)—blank bivalve sample and (**B**)—blank bivalve sample spiked with the 43 compounds at the concentration of the middle validation level as presented in Table 1.

Specificity and selectivity were evaluated by analyzing 20 blank bivalve samples. The inexistence of any interference able to compromise the accurate identification of the target antibiotics along with the unequivocal analysis of the same 20 blank samples spiked at the MRL concentration of all antibiotics, proving the fulfilment of the required specificity and selectivity of σ. Presented in Figure 2, an UHPLC-ToF-MS chromatogram with the detection of exact mass for all the 43 compounds spiked in a blank bivalve sample at the middle concentration of the validation range. For comparison purposes, a blank bivalve sample, without any of the target compounds, is also presented in Figure 2.

Recovery and precision were verified at the MRL/2, MRL and 2MRL concentrations (Table 1). The spiked blank samples were analyzed at those levels, with six replicates each day for 3 different days. Along with the variation of days of analysis, other slight variations were performed to evaluate the influence of those fluctuations in the method and to conclude about the ruggedness. Further to the variation of days, the technician that performed the analysis and reagent lots (acetonitrile, formic acid, EDTA) provided the confidence about the ruggedness of the method. When verifying the inter-day precision, few cases were not among the acceptable values. For instance, norfloxacin, epi-tetracycline and tylosin presented values above 25% at the MRL. On the other hand, for intra-day precision, the worst values were achieved by enoxacin with 24%. Regarding recovery, the lowest values were obtained for nafcillin with values of 44.1% and 29.8% for MRL and 2MRL, respectively. Despite those, the lowest values were 70% for doxycycline at 1/2MRL and 76.3% for 1/2MRL of cefoperazone. The highest recovery was calculated for nalidixic acid with 124.1% at the MRL concentration.

### 2.2. Application to Real Samples

To evaluate the applicability of the UHPLC-ToF-MS-validated method, it was applied to 48 bivalves intended for human consumption. Only four samples (8.3%) were found to be contaminated. From these, three were frozen commercially acquired samples and one was a wild sample collected in the Albufeira lagoon.

Four antibiotics were found, alone, in these four samples, namely trimethoprim, doxycycline, oxytetracycline and valnemulin. The highest concentration was found for trimethoprim, 165.30 µg kg^−^^1^ (a value higher than the considered MRL 50 µg kg^−^^1^) in a frozen sample of cockles (Cerastoderma edule) originating from the Northeast Atlantic Ocean, North Sea. Doxycycline was found at 7.63 µg kg^−^^1^, also in a cockle sample with similar characteristics. Oxytetracycline was found in a wild clam sample (Ruditapes decussatus) from Portugal (Albufeira lagoon) at 12.48 µg kg^−^^1^. Finally, valnemulin was present in a frozen commercially acquired clam sample (Paratapes undulatus), with an origin in the Midwest Pacific Ocean, at 7.63 µg kg^−^^1^. One should note that, to achieve a full assessment and monitoring, further studies are required including a large number of bivalve samples. In addition, these results highlight the low frequency and contamination level presented in these samples and, although one sample presented a concentration for trimethoprim higher than the MRL, low risk might be expected from this food exposure. However, other types of food, such as fish, meat and fruits, can also contain antibiotics and contribute to antibiotic ingestion through food. However, the results demonstrate that the four positive samples originated from different parts of the globe, suggesting that this type of contamination is widespread. This raises the issue of the emergence of bacterial resistance due to the presence of antibiotics in water, and the importance of biomarkers, such as bivalves, to control this subject. In this particular case, bacterial resistance can be acquired by bacteria, additionally to the gene transfer, due to the low concentration in the water, bivalves or humans (through bivalve ingestion) [23].

In general, in our study, the levels found are in agreement with those of other studies reported in the scientific literature. As previously reviewed, except for oxytetracycline in bivalves belonging to the North Adriatic Sea, all the studies revealed antibiotic residues under the MRLs defined by the competent authorities [8].

Fifteen pharmaceuticals, including three antibiotics, namely ronidazole, sulfamethazaxol and azithromycin, were found in 3 bivalve species from the delta of the Ebro river. These antibiotics ranged from levels lower than the LoQ for sulfamethazaxole in Crossastrea gigas to 3.0 ± 0.1 µg kg^−^^1^ of azithromycin in the same species [12]. Alvarez-Munoz et al. also observed that four antibiotics, out of seven included in the analytical method, were detected in bivalves, namely azithromycin, dimetridazole, sulfamethoxazole and ronidazole. Azithromycin was present in all analyzed samples (n = 50) and its concentration ranged from 1.3 ng/g dw in clams (C. gallina, Ebro delta) to 13.3 µg kg^−^^1^ dw in mussels (M. galloprovincialies, Po Delta). The maximum concentration measured corresponds to samples from the Po Delta, but the mussels collected in the Tagus Estuary also had a similar level (11.8 µg kg^−^^1^ dw) [24].

Another study carried out in different areas of the United Nations for Food and Agriculture (FAO) showed that the presence of antibiotics is not significant in bivalves from Spain and the North Adriatic Sea, with levels ranging from 0.55 µg kg^−^^1^ of tetracycline in mussel harvested in Atlantic Spain to 125.03 µg kg^−^^1^ of oxytetracycline in clam from the North Adriatic Sea. The latter was the only sample that contained a concentration slightly higher than the European Union MRL established for fish [25].

## 3. Materials and Methods

### 3.1. Sampling

A total of 48 samples of bivalves (mussels, clams, cockles and razor clams) intended for human consumption were collected between May 2020 and April 2021. From these, 18 samples were sampled from 4 locations along the Portuguese Atlantic coast (Sado Estuary, Albufeira Lagoon, Ria Aveiro and Matosinhos), while 30 frozen samples were commercially acquired as available for regular consumers from different commercial surfaces in Portugal. These samples with an origin from the Pacific and Atlantic Oceans were harvested from aquaculture and the open sea. The information available on the labels was gathered. Samples were thoroughly minced to ensure homogenization. Until the analysis, samples were stored at −18 °C.

### 3.2. Chemicals, Reagents and Standard Solutions

The analytical standards of the targeted antibiotics, with purity ≥98%, were obtained from Sigma Chemicals Co. (St. Louis, MO, USA). HPLC-grade acetonitrile and methanol were also obtained from Sigma Chemicals Co. (St. Louis, MO, USA). EDTA at 0.1 M was from Honeywell-Riedel-De Haën, Seelze, Germany and n-hexane was from Carlo Erba Reagenti, Milan, Italy. Bi-distilled water was obtained daily through a Milli-Q system (Millipore, Bedford, MA, USA). Formic acid was purchased from Merck (Darmstadt, Germany).

Standard stock solutions, including the internal standard (IS), were prepared with the concentration of 1 mg mL^−^^1^ by weighing the precise amount and diluting in 10 mL of methanol, except for the penicillin and cephalosporins, which were prepared in water for stability reasons. These stock solutions were stored for 6 months at −20 °C and the appropriate dilutions were made to obtain a final mixture working solution to be used at the necessary spiking levels for validation. The same approach was followed for the preparation of sulfameter, the IS working solution, with 10 µg mL^−^^1^. Matrix-matched calibration curves were based on spiked blank samples at concentrations from the maximum residue level (MRL)/5 and 4MRL. The process was performed prior to the sample-extraction procedure.

### 3.3. Sample Extraction

Firstly, 2.0 ± 0.05 g of the homogenized sample was weighed, to which 20 µL of the internal working standard solution was added. The sample was vortexed for 15 s. After resting, sheltered from light, for about 10 min, 10 mL of acetonitrile and 1 mL of 0.1 M EDTA solution were added and vortexed for 15 s. After homogenization in a vertical shaker (Agitelec, J. Toulemonde, Paris, France) for 20 min, a centrifugation step followed at 2879× *g* for 10 min at 4 °C (3–16 K, SIGMA, St. Louis, MO, USA). The supernatant was transferred to a new tube. Two milliliters of n-hexane was added, and the sample was vortexed for 30 *s*, and centrifuged at 2879× *g* for 10 min at 4 °C. The n-hexane phase was discarded and the remaining acetonitrile phase was evaporated to about 0.5 mL.

### 3.4. UHPLC-ToF-MS Analysis

After extraction, the chromatographic analysis was performed using an UHPLC system Shimadzu Nexere X2 coupled to high-resolution mass spectrometry with a time-of-flight analyzer (ToF-MS) 5600 from Sciex (Sciex, Foster City, CA, USA). A Waters Acquity UPLC HSS T3 1.8 μm, 2.1 × 100 mm (Dublin, Ireland) chromatographic column was used and maintained at a temperature of 40 °C.

The final extract of 0.5 mL was added of 0.5 0 mL of 0.1% formic acid (mobile phase A) and filtered, and 10 µL was injected in the system with a flow rate of 0.5 mL/min and a gradient of 0.1% formic acid (A) and acetonitrile (B), as shown in Table 2. Mass spectrometry was performed in an ionization mode with a positive electrospray and the acquisition in a full-scan mode within a mass range of 100–920 Da. In Table 3, the detection conditions for each compound are presented. The acquisition was performed by the software Analyst^®^ TF (Sciex) and the data analysis and processing of results through PeakViewTM, LibraryViewTM and MultiQuantTM (Sciex).

The identification criteria were mainly based on the exact mass accuracy and the relative retention time (RRT) deviation, as described in the Commission Implementing Regulation 2021/808 [20]. For the first parameter, the maximum variation acceptable was 5 ppm, obtained with Equation (4).

Equation (4):

(4) $$\Delta \mathrm{ppm}=\left(\frac{\mathrm{Exact}\;\mathrm{mass}-\mathrm{Mass}\;\mathrm{detected}}{\mathrm{Exact}\;\mathrm{mass}}\right)\times 10^{6}$$

For the variation of the RRT, the acceptance criterion was a maximum of 1% variation, being these values calculated as indicated by Equation (5).

Equation (5):

(5) $$\Delta \mathrm{RRT}\;\left(\%\right)=\left(\frac{{\mathrm{RRT}}_{\mathrm{sample}}-{\mathrm{RRT}}_{\mathrm{standard}}}{{\mathrm{RRT}}_{\mathrm{standard}}}\right)\times 100$$

### 3.5. Method Validation

The method was fully validated in accordance with the CIR 808/2021 [21] to assess: specificity, selectivity, precision, recovery, ruggedness, linearity and the decision limit CCα, and for LoD and LoQ calculations, the ICH guidelines were followed [23]. To minimize the number of samples to be analyzed, a combination of experiments was performed on three different days. The selectivity and specificity were assessed by analyzing 20 different blank bivalve samples and that analysis was performed on three different days. Spiked blank samples were used to build calibration curves in ranges of concentrations for each compound, as presented in Table 1. For the precision and recovery evaluation, six analysis replicates of three levels of concentration (Table 1) were performed on each of the three days. As previously described, the peak areas of both the target antibiotic and internal standard were measured, and all calculations were performed through the ratio of analyte/internal standard areas. The data obtained in the described assays were used to evaluate the parameters needed for the complete validation and by using the presented Equations (1)–(3).

## 4. Conclusions

As demonstrated in the analytical procedure described herein, acetonitrile, EDTA and n-hexane extraction through homogenization and centrifugation allowed for the simultaneous, rapid and sensitive detection and quantification of 43 antibiotics in bivalve samples. This allowed us to use these organisms as a tool for environmental monitoring, to evaluate the eventual risk to human health following consumption of these filter feeders and to assure that the maximum limits established by the EU legislation are complied with.

The results of 48 collected bivalve samples revealed low detection frequencies and concentrations below the MRLs defined, with the exception for trimethoprim in one sample.

To achieve a full assessment and monitoring, further studies are required with a large number of food samples and to verify the effects of cooking procedures. The risk for consumers lies in a direct or indirect effect through the potential antimicrobial resistance mediated by the presence of antibiotics. This risk has to be evaluated considering that bivalves are normally cooked before being consumed. However, little is known about the effects of these treatments on pharmaceutical residues, namely antibiotics.

## Figures and Tables

**Table 1:** Summary of the analytical quality parameters evaluated for method validation.

	Spiking Level	Recovery	Precision		Linearity (*r*^2^)	LoD	LoQ	Ccalfa	Max Δppm	U (%)
	(µg kg^−1^)	(%)	Intra-Day RSD (%)	Inter-Day RSD (%)		(µg kg^−1^)	(µg kg^−1^)	(µg kg^−1^)		
Amoxicillin	25	116.4	16.4	12.6	0.9995	3.31	10.00	63.67	1.99	20
	50	119.5	14.8	22.6						
	100	96.9	11.7	17.9						
Ampicillin	25	104.9	9.8	14.8	0.9987	6.38	19.30	61.01	1.60	21
	50	109.9	14.5	22.1						
	100	90.5	9.4	14.4						
Benzylpenicillin	25	83.2	14.1	14.1	0.9922	11.80	35.80	66.23	−1.20	26
	50	85.2	19.8	19.8						
	100	80.7	13.9	13.9						
Cefalonium	10	81.4	8.7	13.2	0.9835	3.05	9.7	24.12	1.21	18
	20	99.5	15.1	23.0						
	40	94.9	8.8	13.5						
Cefapirin	25	83.9	10.7	16.2	0.9912	11.90	36.20	61.28	0.33	20
	50	94.1	16.4	24.9						
	100	84.3	9.7	14.7						
Cefazolin	25	77.6	10.1	15.4	0.9950	8.81	26.70	61.82	2.56	18
	50	82.2	13.6	20.8						
	100	78.6	10.1	15.4						
Cefoperazon	25	76.3	9.0	13.6	0.9902	7.48	22.70	62.25	−1.30	16
	50	90.6	11.3	17.1						
	100	84.5	10.5	16.0						
Cefquinome	25	82.5	8.9	13.5	0.9934	11.50	34.90	62.00	−3.22	20
	50	91.8	15.5	23.6						
	100	84.2	10.3	15.7						
Cephalexin	25	115.5	12.7	19.4	0.9957	8.20	24.80	62.83	1.55	22
	50	116.4	15.2	23.1						
	100	91.6	11.0	16.8						
Chlortetracycline	50	100.0	9.1	13.8	0.9983	10.40	31.50	121.73	2.71	15
	100	101.2	15.9	24.1						
	200	84.3	9.3	14.2						
Cinoxacin	50	112.5	11.7	17.9	0.9953	17.10	51.90	122.35	−1.20	21
	100	109.6	14.8	22.5						
	200	83.8	9.6	14.6						
Ciprofloxacin	50	103.8	10.0	15.2	0.9987	8.92	27.00	124.37	−0.99	17
	100	100.5	16.1	24.5						
	200	84.8	10.5	15.9						
Danofloxacin	50	91.3	8.9	13.6	0.9993	6.62	20.10	121.98	1.26	21
	100	94.4	14.5	22.5						
	200	80.6	9.4	14.4						
Dicloxacillin	150	104.7	14.1	21.4	0.9929	45.00	136.00	380.97	3.01	21
	300	108.0	15.4	23.4						
	600	98.1	11.6	17.6						
Doxycycline	50	70.0	13.2	20.1	0.9927	22.60	68.60	133.49	1.32	21
	100	76.0	14.9	22.6						
	200	84.4	14.4	21.9						
Enoxacin	50	95.9	13.8	13.8	0.9994	6.16	18.70	133.93	1.54	14
	100	93.5	24.0	24.4						
	200	76.8	14.6	14.6						
Enrofloxacin	50	107.5	10.7	16.3	0.9994	6.16	18.70	125.73	−3.01	20
	100	112.3	15.5	23.6						
	200	91.8	11.0	16.8						
epi-Chlortetracycline	50	83.8	9.2	13.9	0.9981	11.10	33.30	124.95	−2.11	18
	100	88.7	15.4	23.4						
	200	78.6	10.7	16.3						
epi-Tetracyclin	50	95.0	15.5	23.6	0.9717	11.90	36.40	135.23	2.21	23
	100	97.2	18.3	27.8						
	200	76.8	15.1	23.0						
Flumequine	100	88.7	10.3	15.7	0.9958	30.70	93.20	251.20	3.04	22
	200	93.4	12.0	18.3						
	400	83.4	11.0	16.7						
Marbofloxacin	75	95.1	9.1	13.9	0.9983	15.50	47.10	188.32	0.34	15
	150	98.4	15.0	22.9						
	300	80.2	11.0	16.7						
Nafcillin	150	81.2	1.9	2.9	0.9943	54.10	164.00	315.52	0.47	6
	300	44.1	2.4	3.6						
	600	29.8	2.2	3.4						
Nalidixic acid	50	106.5	10.9	16.5	0.9961	15.60	47.40	124.09	−0.88	2214
	100	124.1	16.0	24.4						
	200	92.4	10.3	15.7						
Norfloxacin	50	97.9	9.6	14.6	0.9920	7.10	21.50	122.53	−0.23	16
	100	98.3	16.6	25.2						
	200	82.7	9.7	14.7						
Ofloxacin	50	97.8	10.0	15.2	0.9983	10.30	31.10	123.62	1.51	22
	100	99.6	16.0	24.4						
	200	82.1	10.1	15.4						
Oxacillin	150	86.8	10.5	15.9	0.9922	57.90	175.00	363.22	2.09	20
	300	94.9	13.5	20.5						
	600	82.3	9.0	13.8						
Oxolinic acid	150	123.1	9.1	13.9	0.9958	45.80	139.00	365.37	0.83	18
	300	113.3	13.1	20.0						
	600	88.7	9.4	14.2						
Oxytetracycline	50	86.1	6.7	10.3	0.9931	20.80	63.10	128.73	−2.43	16
	100	82.7	11.5	17.4						
	200	79.0	12.3	18.8						
Sulfachloropyridazine	50	97.7	8.7	13.2	0.9962	14.70	44.60	135.71	1.61	20
	100	85.2	15.1	23.0						
	200	92.0	15.3	23.3						
Sulfadiazine	50	112.6	8.4	12.8	0.9970	12.90	39.10	114.93	1.56	11
	100	105.1	7.6	11.6						
	200	89.9	6.4	9.8						
Sulfadimethoxine	50	77.9	11.2	17.1	0.9972	12.60	38.20	132.49	1.09	20
	100	78.2	6.7	10.2						
	200	87.6	13.9	21.2						
Sulfadimidin	50	113.8	6.4	9.7	0.9953	16.10	48.70	116.96	−0.98	11
	100	104.4	4.7	7.2						
	200	92.1	7.3	11.1						
Sulfadoxine	50	116.6	5.0	7.6	0.9984	9.50	28.80	121.42	−0.96	12
	100	107.4	4.6	7.0						
	200	92.6	9.2	14.0						
Sulfapyridin	50	115.6	6.0	9.1	0.9984	9.42	28.50	118.25	1.29	17
	100	109.3	7.0	10.7						
	200	92.8	7.8	11.9						
Sulfaquinoxaline	50	77.2	13.4	20.4	0.9902	23.80	72.00	136.85	−0.89	22
	100	91.0	9.0	13.8						
	200	80.1	15.8	24.1						
Sulfathiazole	50	77.6	10.2	15.6	0.9953	16.10	48.70	126.70	1.77	15
	100	82.4	14.1	21.5						
	200	87.4	11.5	17.4						
Sulfisomidine	50	109.1	6.7	10.2	0.9929	19.80	60.00	115.85	−0.69	13
	100	109.6	10.0	15.2						
	200	89.6	6.8	10.4						
Sulfisoxazole	50	115.3	8.1	12.4	0.9981	10.30	31.10	130.27	−0.99	20
	100	107.2	11.2	17.0						
	200	103.7	13.0	19.8						
Tetracycline	50	95.1	8.7	13.3	0.9975	4.43	13.40	129.85	1.79	18
	100	77.5	15.2	23.2						
	200	90.7	12.8	19.5						
Tilmicosin	25	92.6	12.6	19.1	0.9969	7.11	21.60	60.92	1.91	15
	50	93.6	15.1	22.9						
	100	77.8	9.4	14.3						
Trimethoprim	25	108.7	9.0	13.7	0.9950	8.28	25.10	59.98	−0.93	19
	50	106.1	12.7	19.3						
	100	83.4	8.6	13.0						
Tylosin A	50	93.4	12.5	19.1	0.9978	11.30	34.10	121.90	0.98	15
	100	97.3	17.0	25.9						
	200	83.9	9.4	14.3						
Valnemulin	25	85.0	15.4	23.4	0.9990	4.15	12.60	65.18	1.85	22
	50	96.9	16.3	24.8						
	100	86.7	13.0	19.8						

**Table 2:** Gradient elution scheme.

Time (min)	% A	% B
0	97	3
2	97	3
5	40	60
9	0	100
10	97	3
11	97	3

**Table 3:** MS conditions for each compound.

Antibiotic	Molecular	Mass (Da)	[M+H]^+^ (Da)	RT (min)
	Formula			
Amoxicillin	C_16_H_19_N_3_O_5_S	365.10454	366.11182	3.6
Ampicillin	C_16_H_19_N_3_O_4_S	349.10963	350.11690	4.2
Benzylpenicillin	C_16_H_18_N_2_O_4_S	334.09873	335.10601	4.4
Cefalonium	C_20_H_18_N_4_O_5_S_2_	458.5110	459.5180	4.1
Cefapirin	C_17_H_17_N_3_O_6_S_2_	423.05588	424.06316	4.0
Cefazolin	C_14_H_14_N_8_O_4_S_3_	454.03002	455.03729	4.6
Cefoperazon	C_25_H_27_N_9_O_8_S_2_	645.14240	646.14968	4.9
Cefquinome	C_23_H_24_N_6_O_5_S_2_	528.12496	529.13224	3.9
Cephalexin	C_16_H_17_N_3_O_4_S	347.09398	348.10125	4.2
Chlortetracycline	C_22_H_23_ClN_2_O_8_	478.11429	479.12157	4.6
Cinoxacin	C_12_H_10_N_2_O_5_	262.05897	263.06625	5.0
Ciprofloxacin	C_17_H_18_FN_3_O_3_	331.13322	332.14050	4.4
Danofloxacin	C_19_H_20_FN_3_O_3_	357.14887	358.15615	4.4
Dicloxacillin	C_19_H_17_Cl_2_N_3_O_5_S	469.02660	470.03387	6.2
Doxycycline	C_22_H_24_N_2_O_8_	444.15327	445.16054	4.9
Enoxacin	C_15_H_17_FN_4_O_3_	320.12847	321.13575	4.3
Enrofloxacin	C_19_H_22_FN_3_O_3_	359.16452	360.17180	4.5
epi-Chlortetracyclin	C_22_H_23_ClN_2_O_8_	478.11429	479.12157	4.4
epi-Tetracyclin	C_22_H_24_N_2_O_8_	444.15327	445.16054	4.3
Flumequine	C_14_H_12_FNO_3_	261.08012	262.08740	5.7
Marbofloxacin	C_17_H_19_FN_4_O_4_	362.13903	363.14631	4.3
Nafcillin	C_21_H_22_N_2_O_5_S	414.12494	415.13222	6.0
Nalidixic acid	C_12_H_12_N_2_O_3_	232.08479	233.09207	5.6
Norfloxacin	C_16_H_18_FN_3_O_3_	319.13322	320.14050	4.3
Ofloxacin	C_18_H_20_FN_3_O_4_	361.14378	362.15106	4.3
Oxacillin	C_19_H_19_N_3_O_5_S	401.10454	402.11182	5.8
Oxolinic acid	C_13_H_11_NO_5_	261.06372	262.07100	5.2
Oxytetracycline	C_22_H_24_N_2_O_9_	460.14818	461.15546	4.1
Sulfachloropyridazine	C_10_H_9_ClN_4_O_2_S	284.01348	285.02075	4.9
Sulfadiazine	C_10_H_10_N_4_O_2_S	250.05245	251.05972	4.0
Sulfadimethoxine	C_12_H_14_N_4_O_4_S	310.07358	311.08085	5.3
Sulfadimidin	C_12_H_14_N_4_O_2_S	278.08375	279.09102	4.6
Sulfadoxine	C_12_H_14_N_4_O_4_S	310.07358	311.08085	5.0
Sulfapyridin	C_11_H_11_N_3_O_2_S	249.05720	250.06447	4.2
Sulfaquinoxaline	C_14_H_12_N_4_O_2_S	300.06810	301.07537	5.3
Sulfathiazole	C_9_H_9_N_3_O_2_S_2_	255.01362	256.02090	4.2
Sulfisomidine	C_12_H_14_N_4_O_2_S	278.08375	279.09102	3.9
Sulfisoxazole	C_11_H_13_N_3_O_3_S	267.06776	268.07504	5.1
Tetracycline	C_22_H_24_N_2_O_8_	444.15327	445.16054	4.5
Tilmicosin	C_46_H_80_N_2_O_13_	868.56604	869.57332	4.9
Trimethoprim	C_14_H_18_N_4_O_3_	290.13789	291.14517	4.3
Tylosin A	C_46_H_77_NO_17_	915.51915	916.52643	5.3
Valnemulin	C_31_H_52_N_2_O_5_S	564.35970	565.36697	5.6

## Data Availability

The data presented in this study are available on request from the corresponding author.
